# Easing anxiety symptoms through leisure activities during social isolation: Findings from nationally representative samples

**DOI:** 10.1371/journal.pone.0303585

**Published:** 2024-06-10

**Authors:** Queena Cheong, Arminee Kazanjian, Joseph H. Puyat

**Affiliations:** 1 Department of Occupational Therapy, Faculty of Medicine, University of Toronto, Toronto, Ontario, Canada; 2 School of Population & Public Health, The University of British Columbia, Vancouver, Canada; 3 Centre for Advancing Health Outcomes, Providence Health Care, British Columbia, Vancouver, Canada; King Khalid University, EGYPT

## Abstract

Public health interventions implemented during the COVID-19 pandemic may exacerbate anxiety symptoms for many. We conducted this study to better understand the role of leisure activity in promoting mental wellness during times of social isolation and reduced access to recreation facilities and mental health support services. We analyzed nationally representative survey data collected by Statistics Canada as part of the Canadian Perspectives Survey Series (CPSS) during May 4–10 (CPSS 2) and July 20 to 26, 2020 (CPSS 4). Data related to leisure activity and anxiety symptoms as measured by a score of more than 10 on the General Anxiety Disorder scale were examined using descriptive and log-binomial regression analyses. Survey sampling weights were applied in all analyses, and regression results were adjusted for sociodemographic characteristics. Exercise and communication with friends and loved ones were the most frequently reported leisure activity. Prevalence of moderate to severe anxiety symptoms reported by participants was lower in CPSS 4 compared to CPSS 2. Results of adjusted log-binomial regression analyses revealed lower prevalence of moderate to severe anxiety symptoms in those who engaged in exercise and communication, while those who meditated exhibited higher prevalence. In conclusion, leisure activities, such as exercise and communication with loved ones, can promote mental wellness. Future research should clarify the role of meditation for mental wellness promotion during periods of social isolation.

## Introduction

The COVID-19 pandemic brought upon mass implementation of public health directives to social distance, self-isolate, and quarantine. While physically keeping apart two meters from the nearest person or staying at home to separate from loved ones after being exposed to an infectious agent may be beneficial for reducing overall community disease transmission, it may not be for mental health [1–3].

In March 2020, Canada declared the COVID-19 pandemic as a public health emergency and implemented many public health measures as a response to contain the spread of the infectious disease. Social distancing is found to be one of the most reliable responses to avoid the spread of the coronavirus, thus leading to the nationwide closure of many in-person social support services and cancellation of recreation programs to avoid contact between participants [4, 5]. Recreation programs are sources of mental health promotion for many, and these restrictions meant that Canadians were unable to access spaces for mental health support for several months.

With an increase of social distancing and lockdown measures being implemented during this time, researchers and Canadian psychiatric organizations have found that anxiety is one of the most frequently reported mental health symptoms in Canada during the COVID-19 pandemic [6, 7]. Defined by the Diagnostic and Statistical Manual of Mental Disorders (DSM-5), anxiety is a state characterized by excessive worry or fear that has been present for more days than not for the past six months [8]. The nationwide effects on anxiety symptoms of the COVID-19 pandemic in Canada are documented by various government agencies and research studies. A survey by Statistics Canada reported that 46,000 Canadians reported poorer mental health since the onset of social distancing, and that 88% had experienced at least one symptom of anxiety during the COVID-19 pandemic in 2020 [9]. Research conducted by Sylvestre et al. found that symptoms of anxiety increased significantly among Canadian young adults during the COVID-19 pandemic [10].

Published studies have reported on the effectiveness of leisure activities for anxiety symptoms [11]. Leisure activities are defined as the activities that individuals perform outside of everyday responsibilities to relax, and tend to reflect individuals’ social systems [12]. Performing activities such as physical activities, meditation, or communication with friends have been found to reduce anxiety and ease worry. Proposed explanations for leisure activities’ role in promoting mental health include the activities’ ability to provide individuals with an elevated sense of social inclusiveness [13], sense of achievement, and meaning [14].

Leisure activities also reflect individuals’ social systems. Previous studies have underscored the significance of social relationships in the leisure experience, particularly as a factor that moderates the impact of leisure activity on anxiety. For example, performing physical activity with a group rather than individually has been found to increase well-being and decrease anxiety [15]. Additionally, doing leisure activities with other persons may achieve additional positive effects compared to if performed alone [16–18]. This is worth noting as such conclusions were drawn from data collected from pre-COVID times where leisure facilities were more accessible to facilitate social relationships. There exist only a few studies that examine leisure activities in the context of events with limited opportunities for social contact or support. Furthermore, there are limited studies that examine the effects of leisure activities on anxiety during the COVID-19 pandemic in a Canadian population. Thus, the current study aims to fill in existing knowledge gaps by performing secondary analysis on nationwide data to examine how leisure activities performed in individual settings and without social support systems can influence anxiety symptoms during the COVID-19 pandemic.

## Materials and methods

### Data source

We examined longitudinal cross-sectional survey data collected by Statistics Canada during the COVID-19 pandemic in Canadian Perspectives Survey Series (CPSS) 2 (n = 4,600) [19]; collected from May 4 to 10, 2020) and CPSS 4 (n = 4,218; July 20 to 26, 2020) [20]. The CPSS was an initiative aimed at understanding the impacts of the COVID-19 pandemic on Canadians and their health behaviors. Data from CPSS 1, 3, 5, and 6 were excluded, as they did not collect information on either leisure activities or anxiety symptoms.

The Public Use Microdata File version of the CPSS data were accessed through the University of British Columbia’s (UBC) data library; ethics approval was covered by the secondary use of anonymous information clause (Item 7.10.4) of UBC Policy #LR9 [21].

### Measures

Among those who did and did not engage in leisure activities (communication with friends and family, meditation, exercise), we examined the percentage of those who self-reported experiencing moderate to severe anxiety (>10 GAD-7 scores). Those who reported engaging in the activities did so for either mental health, physical health, or for both mental and physical health reasons. We applied the survey sampling weights provided by Statistics Canada to describe the sample according to sociodemographic characteristics that were reported in CPSS 2 and 4. The measured sociodemographic variables included sex, age, educational attainment, marital status, household size and rural-urban classification.

### Statistical analyses

To examine the association between leisure activity engagement and prevalence of moderate to severe anxiety symptoms, we ran log-binomial regression analyses with and without adjustment for socio-demographic characteristics that could influence engagement in the activities of interest and anxiety levels. These regression analyses generate ratios that describe the prevalence of moderate to severe anxiety disorders in those that engaged in the activities compared with those that did not perform the activities as the reference group. A prevalence ratio less than one indicates that performing the activity was associated with lower prevalence of moderate to severe anxiety symptoms, while a prevalence ratio above one suggests the opposite. All descriptive and regression analyses used the sampling weights provided by Statistics Canada. Stata Version 16.1 was used to generate all statistical outputs.

## Results

There were 4,600 and 4,218 respondents that participated in the CPSS 2 and 4. In both surveys, majority of the sample were between the ages of 35 and 64, had post-secondary education, employed, married, lived in a household with more than one person, and resided in urban areas. There were equal proportions of males and females in both surveys (Table 1).

**Table 1:** Sample characteristics, Canadian Perspective Survey Series 2 and 4.

		CPSS Series 2 (May 4–10, 2020)		CPSS Series 4 (July 20–26, 2020)	
		Count	Weighted %	Count	Weighted %
Total		4600	100.0	4218	100.0
Sex					
	Male	2116	49.4	1944	49.4
	Female	2484	50.7	2274	50.6
Age					
	15 to 34	845	31.0	687	31.0
	35 to 64	2542	47.9	2329	47.9
	65 and older	1213	21.1	1202	21.2
Education					
	High school or lower	1145	40.0	1019	39.0
	Trade, some college/university	1687	31.5	1538	32.2
	Bachelor’s degree and higher	1768	28.5	1661	28.7
Employment Status					
	Employed	2676	55.3	2424	58.4
	Unemployed	1890	44.0	1708	38.6
	Missing	34	0.7	86	3.0
Marital Status					
	Married	2338	51.5	2177	49.8
	Living common-law	498	11.3	442	11.1
	Widowed/Separated/Divorced	758	10.2	722	11.4
	Single, never married	1006	27.0	877	27.7
Household Size					
	1	1320	15.4	1214	15.6
	2	2602	51.6	2416	52.8
	3 and more	678	33.0	588	31.6
Rural/Urban					
	Rural	975	16.3	878	15.7
	Urban	3625	83.7	3340	84.3

Note: Percentages were generated using sampling weights provided by Statistics Canada.

Of the three activities examined, exercise (84.8% in CPSS 2 and 85.2% in Series 4) and communication with friends and family (90.9% and 91.2% in CPSS 2 and 4, respectively) were the two activities that had the highest level of participation. In contrast, about 23.6% (CPSS 2) and 25.4% (CPSS 4) practiced meditation.

Overall, the proportion of respondents that met the criteria for moderate to severe anxiety, as measured by a GAD-7 cut-point of 10, was higher in CPSS 2 (18.1%) than in CPSS 4 (13.1%). By type of activity, the descriptive results indicate that the prevalence of moderate to severe anxiety was lower among those that engaged in exercise or communicated with friends and family, but higher among those that engaged in meditation (Table 2). This trend was present in both CPSS 2 and 4.

**Table 2:** Association between exercise, meditation and communication with friends and family and prevalence of moderate to severe anxiety.

		Performed the Activity, Column %	Prevalence of Moderate to Severe Anxiety, Row %	Moderate/Severe Anxiety Prevalence Ratio	
				Unadjusted	Adjusted
*CPSS Series 2* *(May 4–10*, *2020)*					
Total Sample		-	18.1	-	-
Exercise					
	Yes	84.8	17.0	**0.69 (0.51, 0.95)**	0.76 (0.53, 1.06)
	No	15.2	24.4	Reference	Reference
Meditation					
	Yes	23.6	24.0	**1.47 (1.14, 1.89)**	**1.43 (1.10, 1.85)**
	No	76.4	16.4	Reference	Reference
Communication					
	Yes	90.9	18.0	0.91 (0.61, 1.33)	0.83 (0.55, 1.25)
	No	9.1	19.9	Reference	Reference
*CPSS Series 4* *(July 20–26*, *2020)*					
Total Sample		-	13.1	-	-
Exercise					
	Yes	85.2	12.0	**0.62 (0.43, 0.89)**	**0.59 (0.40, 0.86)**
	No	14.9	19.5	Reference	Reference
Meditation					
	Yes	25.4	17.7	**1.52 (1.12, 2.04)**	**1.61 (1.10, 2.33)**
	No	74.6	11.7	Reference	Reference
Communication					
	Yes	91.2	12.7	0.65 (0.35, 1.20)	**0.59 (0.35, 0.99)**
	No	8.8	18.3	Reference	Reference

Note: Column and row percentages are generated using the sampling weights provided by Statistics Canada. Covariates included in estimating adjusted prevalence ratios were sex, age, education, employment status, marital status, household size and residence in rural or urban areas. Prevalence ratios in bold are statistically significant at p < .05.

Results of the log-binomial regression analyses, adjusting for various sociodemographic characteristics, show the same pattern with results based on the CPSS 4 data demonstrating all statistically significant associations (Table 2). The results suggest that the prevalence of moderate to severe anxiety was lower in those that exercised (aPR: 0.59; 95% CI: 0.40–0.86) and in those that communicated with friends and family (aPR: 0.59; 95% CI: 95% CI: 0.35, 0.99). Conversely, the prevalence of moderate to severe anxiety was about 61% higher among those that engaged in meditation compared to those that did not meditate (aPR: 1.61; 95% CI: 1.10, 2.33).

## Discussion

Public health interventions to contain the spread of the COVID-19 virus have disrupted both the daily routines of individuals and operations of many businesses, leading to sharp spikes in anxiety [22]. In this study, we investigated the association between varying types of leisure activities and prevalence of moderate to severe anxiety in May and July 2020 time periods of the COVID-19 pandemic where many lockdown measures were still in effect across the country.

Our results indicate that the general mental health of participants was worse during May compared to July 2020. We also found that there was a greater percentage of moderate to severe anxiety in those who did not exercise or communicate with friends and family, although the associations were only statistically significant during July 2020. In contrast, the percentage of those with moderate to severe anxiety symptoms were lower among those who did not engage in meditation and the association was statistically significant in May and July 2020. Overall, the findings suggest that exercising and communicating with friends and family seemed to provide psychological benefits, whereas meditating did not.

It is no surprise that our findings revealed the effectiveness of exercise in reducing anxiety, as it has been shown that exercise is essential to reducing stress and improving mental health through various physiological and psychological mechanisms. Although it would seem that physical activity (PA) would be low during the pandemic as there is evidence for studies for PA guideline compliance dropping 20% during lockdowns [23] and step counts for 187 countries decreased by more than a quarter on average [24], our findings supported the contrary, with exercising being the second highest activity that participants engaged in for their health at 84.8% and 85.2%.

Interestingly, our analyses indicate that the beneficial effects of exercise for anxiety were only statistically significant during July 2020. This could be attributed to the different restrictions imposed by the Canadian government on the sport and fitness industry during May and July 2020. For nine out of ten provinces in Canada, all indoor and outdoor recreation facilities and activities were suspended at the time CPSS 2 were being collected in May 2020 [25–32]. When data were collected during the mid-week of July 2020 for CPSS 4, all ten provinces eased restrictions on recreation activities, but with varying degrees of restrictions [33, 34]. The recreational landscape in Canada during these two months is critical for the interpretation of our results, as the removal of access to recreation centres and fitness classes would undoubtedly impede exercise routines [5] and the interpersonal relationships gym attendees develop with each other [35]. The immense role that social interaction plays in the fitness experience cannot be emphasized enough, as it has been reported to significantly mediate between many health behaviors and mental health [36]. As well, a study that compared the mental health of 1.2 million survey respondents who did and did not exercise found that those who engaged in popular team sports experienced the greatest reduction in mental health burden compared to the other activities at 22.3% lower [37].

A similar pattern of higher prevalence of moderate to severe anxiety was noted among those who communicated with their friends and family, with such association only being significant during July 2020. This suggests that when anxiety levels are generally high, such as during May 2020 when 18% of the sample had moderate to severe anxiety, communicating with loved ones or with anyone may not have a pronounced effect in reducing one’s anxiety. Individuals experiencing anxious feelings often find it emotionally effortful and challenging to communicate [38], and this could affect both the messages being conveyed and the manner of communication.

An unexpected finding that the current study wishes to underscore is the lower prevalence of anxiety reported among those who did not meditate. This contradicts popular beliefs about meditation as a low risk, beneficial mental wellness promotion, self-regulation, and stress reduction practice [39].

Broadly defined, meditation is a practice that serves to increase mindfulness, a mental state that involves being attentive and aware of the present moment [40]. Much literature has supported the benefits of mindfulness for psychological disorders, as welcoming one’s current experience with embrace, openness, and acceptance can counter maladaptive strategies in dealing with stressors (such as experiential avoidance) [41]; allow for disengagement from ruminative thinking [42], and decrease negative reactivity [43]. Given the benefits of meditation on mental wellness, many engage in meditation to alleviate feelings of anxiety. In 2016, a study found that 94.74% of undergraduate psychology students reported reduction of negative emotional experiences (such as stress, anxiety, panic, and depression) as their reason for beginning meditation, with 95.77% also continuing to meditate for the same reason [44]. Another study found that 37% of 27 young adults reported that their primary reasons for signing up to participate in an intensive Vipassana meditation retreat was to improve self-regulation (which was conceptualized as goals of “becoming more relaxed” and “learning to stop negative thoughts” in the study) [45].

While many who experience anxiety symptoms may have a penchant for meditation, there are also participants who describe feeling anxious after meditating. In 2019, a study reported that participants with higher levels of repetitive negative thinking (RNT) were more likely to experience unpleasant feelings during meditation, whereas female and religious participants were less likely to [39]. In another study, fifty-nine factors were identified to be associated with meditation-related challenges and the study’s notable finding is that medical, psychological, and trauma history influence the presence and duration of such challenges, whereas meditation practice environments (particularly in contexts of social isolation) was commonly described as a risk factor [46]. The study also found that the most frequently reported experience by practitioners (82%) and experts (72%) was “fear, anxiety, panic, or paranoia” [46].

Since our data were collected during the pandemic where the event has been regarded as a traumatic stressor [47] with significant increases in RNT [48], it is possible that increased RNT and trauma and other unpleasant meditation experiences can in part explain the higher prevalence of moderate to severe anxiety in meditators within the current study. Furthermore, since our survey collection occurred in May and July 2020 where indoor social gatherings or indoor recreation settings were still restricted to some degree across the nation, it is possible that a higher percentage of those who meditated within our current study experienced anxiety compared to others because they were doing so from a location of social isolation [46].

The strengths of our study include the secondary analysis of survey data from a nationally representative sample [49]. As well, the two surveys were conducted during the first few months of stay-at-home restrictions as well as after they were relaxed, describing patterns of leisure activities and anxiety symptoms during varying levels of public health restrictions.

While cross-sectional survey data are a cost and time effective method for generating information about prevalence on a population-level [50], our data limit our ability to draw causal inferences and its direction in regard to the association between leisure engagement and anxiety. Furthermore, our study is subject to limitations that are associated with studies that employ self-reported data [51].

## Limitations

The current study utilizes analysis of secondary data, which restricted our analyses to the variables that were available in the data. For example, the specific details of the meditation that participants performed in, such as the technique, type or how often it was practiced, were unclear to us because the database we accessed did not provide this information. As well, we were unable to account for other potential confounders that are not available in the data we examined.

## Conclusion

Our findings highlight the potential benefits of communication and exercise as alternative, home-based methods for mental wellness promotion when access to external facilities, professionals, or resources are limited or immediately possible. As the world navigates the ongoing effects of the pandemic and other circumstances that limit traditional sources of support, communication with loved ones and regular physical activity will play an increasingly important role.

Our analysis also uncovered a potentially negative association between meditation and anxiety symptoms during times of social isolation. Further research is needed to explicate the specific mechanisms by which meditation may amplify anxiety symptoms during social isolation. Additional data about the type of meditation and how it was practiced may help shed light on this counterintuitive association between meditation and anxiety symptoms.
